# Piezoelectric Transducers: Complete Electromechanical Model with Parameter Extraction

**DOI:** 10.3390/s24134367

**Published:** 2024-07-05

**Authors:** Michael L. Isaf, Gabriel A. Rincón-Mora

**Affiliations:** School of Electrical and Computer Engineering, Georgia Institute of Technology, Atlanta, GA 30332, USA; rincon-mora@gatech.edu

**Keywords:** coupling coefficient, electromechanical model, energy harvesting, equivalent impedance, irregular and regular vibrations, mode of vibration, Norton and Thevenin equivalent, parameter extraction, piezoelectric

## Abstract

This paper presents a complete electromechanical (EM) model of piezoelectric transducers (PTs) independent of high or low coupling assumptions, vibration conditions, and geometry. The PT’s spring stiffness is modeled as part of the domain coupling transformer, and the piezoelectric EM coupling coefficient is modeled explicitly as a split inductor transformer. This separates the coupling coefficient from the coefficient used for conversion between mechanical and electrical domains, providing a more insightful understanding of the energy transfers occurring within a PT and allowing for analysis not previously possible. This also illustrates the role the PT’s spring plays in EM energy conversion. The model is analyzed and discussed from a circuits and energy harvesting perspective. Coupling between domains and how loading affects coupled energy are examined. Moreover, simple methods for experimentally extracting model parameters, including the coupling coefficient, are provided to empower engineers to quickly and easily integrate PTs in SPICE simulations for the rapid and improved development of PT interface circuits. The model and parameter extractions are validated by comparing them to the measured response of a physical cantilever-style PT excited by regular and irregular vibrations. In most cases, less than a 5–10% error between measured and simulated responses is observed.

## 1. Introduction

To increase ease of use, practicality, and desirability, many devices, especially in the Internet of Things, require minimal maintenance power methods. Numerous mechanical, biomedical, and civil systems produce vibrations, and piezoelectric transducers (PTs), with their high energy densities and off-the-shelf availability, can effectively and easily tap into this energy source [1,2,3,4,5].

**Figure 1:**
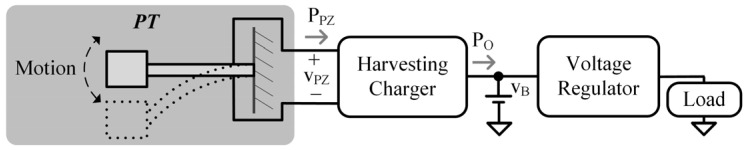
Piezoelectric-powered energy-harvesting system.

PTs convert mechanical energy, in the form of motion or vibration, to electrical energy that can be harvested to charge a battery, $v_{B}$, and power a microsystem, as illustrated in Figure 1. Ultimately, maximizing the harvester’s output power, $P_{O}$, is most important, but to do so, an understanding of how the PT delivers power, $P_{PZ}$, is required [6,7,8].

Transducers are not ideal energy sources, so a source model which accurately represents source impedances and energy conversions is important to design energy harvesting systems well. In a PT, an understanding of how mechanical motion produces electrical energy, electrical loading affects output power, and energy couples between domains is necessary when designing harvesters effectively for specific motion inputs. This requires an insightful PT model with parameters that can effectively approximate the physical device. 

The model shown in [9,10,11] provides a circuit representation of both the mechanical and electrical domains of a PT, along with a method of coupling between the two to capture loading effects. However, the transformer’s coupling coefficient and turns ratio are combined into a single coefficient, which removes insight and analysis potential and can lead to inaccuracies. It is also known that PTs exhibit a dual resonance (parallel and series) behavior that is not captured [12,13,14,15]. In other words, that model does not adequately model PT output impedance. Moreover, mechanical engineers often use mechanical methods, material properties, and/or finite element analysis to determine model parameters, which may be inaccessible to many electrical engineers [9,10,11].

Other models exclude the mechanical domain and approximate $i_{PZ}$ to be proportional to the PT’s tip velocity (for cantilevered PTs). In these cases, the PT is assumed to be very weakly coupled, so the electrical load is said to have negligible effect on the PT’s motion [1,4,7,16,17,18,19,20]. It is not clear though, what constitutes weak coupling or how a PT is determined to be weakly coupled. DC-DC converters using PTs, such as those described in [12,13,14,15], require good impedance models for accurate converter and control design. Such applications often use the Van Dyke model, but this model is lumped, leading to potential error in converter design and PT impedance understanding, and its derivation could be expanded on [21]. This model is typically used for “strongly coupled” PTs, which poses similar ambiguity as before.

This paper presents a complete electromechanical (EM) model for PTs independent of strong or weak coupling assumptions, vibration conditions, and transducer geometries, making it generally applicable to many PT types, sizes, and applications. Because the model details enable insight into the power and energy transfers and losses occurring within PTs, it is particularly useful in energy harvesting contexts. PT impedances and resonances are also analyzed and discussed, exhibiting the model’s use for PT resonators. The model is also reduced to its Norton and Thevenin equivalents, which are applicable in all coupling contexts. Simple methods for experimentally extracting model parameters are presented and discussed to empower designers to quickly and easily integrate PTs in SPICE simulations for the rapid and improved development of PT interface circuits.

## 2. Complete Electromechanical Model

The complete EM model of a PT presented in this section strives to capture and distinguish between the aspects of the PT responsible for motion, energy capture, domain conversion, and electrical behavior. Equivalents of the complete model are also presented for ease of use. The model is developed using a cantilever style PT but is generally valid. 

### 2.1. Complete Model

**Figure 2:**
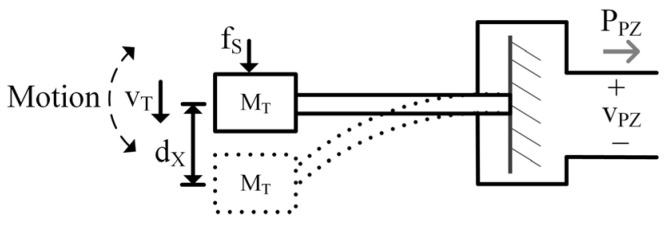
Diagram representing PT motion.

In Figure 2, the PT mass, $M_{T}$, has kinetic energy when in motion with velocity, $v_{T}$. Similarly, a capacitor has energy when a voltage develops across it. So, $M_{T}$ can be modeled as a capacitor, where $C_{T}\equiv M_{T}\;[kg]$, and the mass’s velocity, in $\left[m/s\right]$, is analogous to the capacitor voltage, $v_{T}$ [22,23]:

(1) $$E_{KE}=0.5M_{T}{v_{T}}^{2}\equiv E_{C}=0.5C_{T}{v_{T}}^{2}.$$

A mass moves if force is applied to it and a capacitor develops a voltage if current is applied to it. Since $v_{T}$ represents both voltage and velocity, its time derivative can represent the transducer’s acceleration, $a_{T}$, and since $C_{T}\equiv M_{T}$, the current, $i_{S}$, into $C_{T}$ is analogous to an applied mechanical force, $f_{S}$, on $M_{T}$ in units of $\left[kg\left(m/s^{2}\right)\right]$ or $\left[N\right]$. Therefore,

(2) $$i_{S}=C_{T}\left(\frac{dv_{T}}{dt}\right)\equiv M_{T}a_{T}=f_{S}.$$

Springs store potential energy when compressed (or bent for a cantilever) a distance, $d_{X}$, from rest. Inductors store energy when magnetized by $\phi_{L}$, thus an inductor can model a spring with spring constant, $K_{T}$:

(3) $$E_{PE}=0.5K_{T}{d_{X}}^{2}\equiv E_{L}=0.5\left(\frac{1}{L_{T}}\right){\phi_{L}}^{2}=0.5L_{T}{i_{LT}}^{2},$$

where $L_{T}$ is inversely equal to $K_{T}\;\left[N/m\right]$ and $i_{LT}$ represents force applied by the spring.

Frictional sources, such as air resistance, dissipate energy and dampen mechanical motion in the same way resistors dissipate energy in electrical systems:

(4) $$D_{T}=\frac{f_{D}}{v_{T}}\equiv G_{T}=\frac{1}{R_{T}}=\frac{i_{R}}{v_{T}}.$$

Mechanical dampers, $D_{T}\;\left[kg/s\right]$, apply a force, $f_{D}$, in response to motion, $v_{T}$. So, in keeping with the convention established above, mechanical damping, $D_{T}$, is analogous to the inverse of resistance, $R_{T}$, (i.e., conductance, $G_{T}$) where $i_{R}$ is the current through the resistor and $v_{T}$ is the voltage across it, as seen in (4) [24].

The LC circuit analogous to the spring mass system described above will have the same resonant frequency, $f_{R}$:

(5) $$f_{R}=\frac{1}{2\pi \sqrt{\frac{M_{T}}{K_{T}}}}\equiv f_{LC}=\frac{1}{2\pi \sqrt{L_{T}C_{T}}}.$$

The energy transfer between the mechanical domain (MD) and electrical domain (ED) of a PT is typically modeled with a transformer [9,10,11]. Since PTs and piezoelectric (PZ) materials are not capable of capturing all mechanical energy present for conversion to the ED, a coupling coefficient, $k_{CL}$, exists:

(6) $$k_{CL}\equiv \frac{L_{TC}}{L_{T}}=\frac{L_{TC}}{L_{T0}+L_{TC}}=\frac{C_{LC}’}{C_{L}’}=\frac{C_{LC}’}{C_{L0}’+C_{LC}’}\leq 1.$$

$k_{CL}$ is intrinsic to the PT and is what makes a PT a PT. It represents the inherent strength of the connection between domains—the larger $k_{CL}$, the better the connection. $k_{CL}$ can only be between 0 and 1, inclusive. A $k_{CL}$ of 0 corresponds to a material that is not piezoelectric as this implies no coupling exists between the ED and MD [1,25].

**Figure 3:**
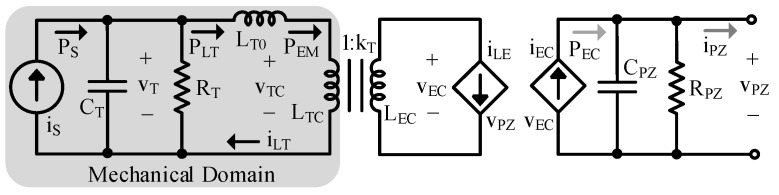
Complete electromechanical PT model.

Figure 3 is the complete EM PT model with the MD modeled on the left. In the state of the art (SoA), $k_{CL}$ is often lumped in with the transformer [9,10,11]. In Figure 3, however, it is represented as the ratio between $L_{TC}$ and $L_{T}$, as (6) shows, where $L_{TC}$ is the portion of $L_{T}$ that perfectly couples with the ED. $k_{CL}$ can also be thought of as the percentage of $v_{T}$ that couples to current in the ED, $i_{EC}$, when the output is shorted.

In a conventional transformer, voltage on the secondary is a multiple of primary voltage by a factor of the turns ratio, $k_{T}$ [26]. Of course, there are no “turns” in a PT, so $k_{T}$ is referred to here as the translation coefficient, representing the conversion between the MD (force/velocity) and the ED (current/voltage):

(7) $$k_{T}\equiv \frac{v_{EC}}{v_{TC}}=\sqrt{\frac{L_{EC}}{L_{TC}}}.$$

In a PT, it is generally accepted that velocity begets current, $i_{EC}$, so the two dependent current sources are used in conjunction with the transformer to couple $v_{T}$ to $i_{EC}$ [1,4,7,9,16,17,18,19,20]. The dependent current sources perform a voltage-to-current conversion which reciprocates impedances (i.e., resistance to conductance, inductance to capacitance, etc.) and dividers (i.e., voltage dividers to current dividers and vice versa). Lastly, $C_{PZ}$ models the PT’s ability to store electrical energy, and $R_{PZ}$ models leakage. $C_{PZ}$ is often dominated by the capacitance that exists between the PT’s electrodes [10,27]. The parameters in Figure 3 are the most fundamental to the model, so they have been italicized in the text to assist the reader in tracking them throughout the paper. 

Presenting the model as it is described here (as opposed to representing mass as inductance and stiffness as capacitance as is often performed in the SoA, such as in [9,10,11]) is vital for a few reasons. First, it allows the inductor to be incorporated into the transformer, illustrating the role the PT stiffness plays in domain coupling/energy transfer by representing the spring as the medium for EM conversion [9]. It also enables explicit visualization of the transformer coupling coefficient by using split inductors to clearly show that only a fraction of the velocity, $v_{TC}$, (i.e., mechanical energy) couples to the ED. Lastly, by using split inductors to represent transformer coupling, the transformer turns ratio can also be explicitly defined. Separating $k_{CL}$ from $k_{T}$ is important because if the coupling coefficient were lumped in with $k_{T}$, it would be incorrectly applied to all impedance translations, as will be shown in Section 2.2. It also introduces the dual resonance behavior into the model, as will be shown in Section 2.3.

This model makes the same assumptions about PTs as those used in and accepted by the SoA, such as in [9,10,11,12,13,14,15,16,17,18,19,20]. These assumptions are: (1) A PT which behaves as a spring-mass-damper system in the mechanical domain is used; (2) Mechanical velocity is in phase with PT short circuit current; (3) The PT’s electrical domain is predominately capacitive. The transformer and split inductors are methods for fundamentally representing conversion and coupling between domains—regardless of its geometry and material, a PT converts energy between domains and only some fraction of energy couples. Therefore, this model and the following analysis can be applied to any PT that satisfies the three assumptions, which encompasses the majority of PTs used in energy harvesting and many PTs used as resonators [1,9,10,11,12,13,14,15,16,17,18,19,20].

### 2.2. Electrical Model

To simplify analysis, it is helpful to use a circuit equivalent to Figure 3 which does not include the transformer or dependent sources. The MD impedances, $Z_{T}$, can be referred to the ED, $Z_{Z}’$, using typical transformer techniques as seen in [26]:

(8) $$Z_{Z}’\equiv Z_{T}{k_{T}}^{2}.$$

As mentioned above, the dependent current sources reciprocate impedances and dividers.

**Figure 4:**
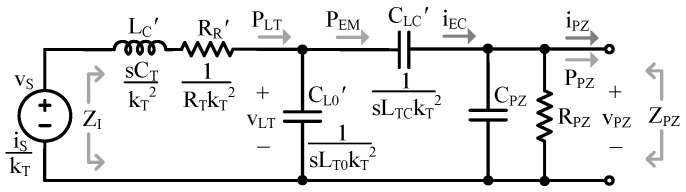
Electrical model.

The results of the above translations are shown in Figure 4. By representing the model this way, coupling between the MD and ED is modeled with a coupling capacitor, $C_{LC}’$, and a leakage capacitor, $C_{L0}’$, which are related to $k_{CL}$ by (6). Note that if $k_{CL}$ and $k_{T}$ were lumped, all translated impedances, instead of just $L_{T0}$ and $L_{TC}$, would be affected by $k_{CL}$. When $k_{CL}$ is greater than 50%, $C_{LC}’$ is larger than $C_{L0}’$, so more than half the mechanical energy present can couple to the ED. When less than 50%, the opposite is true. 

### 2.3. Lumped Electrical Model

**Figure 5:**
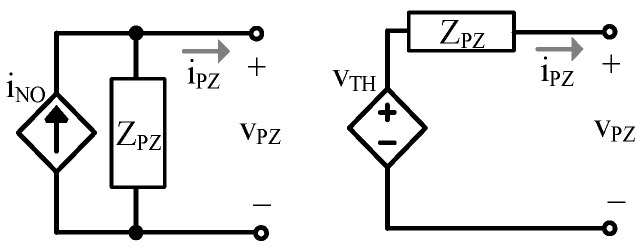
Norton and Thevenin equivalents.

Reducing the model to its Norton and/or Thevenin equivalent, shown in Figure 5, is helpful for simple and easy application. To create these equivalent circuits, an understanding of the PT’s output impedance, $Z_{PZ}$, is required.

$Z_{PZ}$ can be determined by shorting $v_{S}$ in Figure 4 and evaluating impedance when looking left from $v_{PZ}$ [28]:

(9) $$\begin{array}{cc} Z_{PZ} & =R_{PZ}||\frac{1}{sC_{PZ}}||\left(\frac{1}{{k_{T}}^{2}}\left\{\frac{1}{sL_{TC}}+\left[\frac{1}{sL_{T0}}||\left(\frac{1}{R_{T}}+sC_{T}\right)\right]\right\}\right)\\ & =R_{PZ}||\frac{1}{sC_{PZ}}||\left\{\frac{1}{sC_{LC}’}+\left[\frac{1}{sC_{L0}’}||\left(R_{R}’+sL_{C}’\right)\right]\right\}\\ & =R_{PZ}||\left[\frac{1}{s\left(C_{LC}’+C_{PZ}\right)}\right]\left[\frac{{\left(\frac{s}{2\pi f_{R}}\right)}^{2}+\frac{1}{Q_{R}}\left(\frac{s}{2\pi f_{R}}\right)+1}{{\left(\frac{s}{2\pi f_{R}’}\right)}^{2}+\frac{1}{Q_{R}’}\left(\frac{s}{2\pi f_{R}’}\right)+1}\right]\\ & =\left(\frac{R_{PZ}}{1+\frac{s}{2\pi p_{X}}}\right)\left[\frac{{\left(\frac{s}{2\pi f_{R}}\right)}^{2}+\frac{1}{Q_{R}}\left(\frac{s}{2\pi f_{R}}\right)+1}{{\left(\frac{s}{2\pi f_{R}’}\right)}^{2}+\frac{1}{Q_{R}’}\left(\frac{s}{2\pi f_{R}’}\right)+1}\right]. \end{array}$$

At very low frequencies, $L_{C}’$ is effectively a short relative to $C_{LC}’$ and $C_{L0}’$. As frequency increases though, its impedance will no longer be negligible, and it will interact with $C_{LC}’$ and $C_{L0}’$, resulting in a mechanical, series resonance, $f_{R}$, given in (5). The quality factor, $Q_{R}$, at $f_{R}$ is:

(10) $$Q_{R}=\frac{1}{R_{R}’}\sqrt{\frac{L_{C}’}{C_{L}’}}=R_{T}\sqrt{\frac{C_{T}}{L_{T}}}.$$

Above, $f_{R}$, $L_{C}’$ shunts the surrounding capacitors until frequency increases enough for $L_{C}’$ to effectively open relative to them, resulting in an EM, parallel resonance, $f_{R}’$:

(11) $$\begin{array}{cc} f_{R}^{’} & =\frac{1}{2\pi \sqrt{L_{C}’\left[C_{L0}’+(C_{LC}’⊕C_{PZ})\right]}}\\ & =\frac{1}{2\pi \sqrt{C_{T}\left[L_{T0}+\left(L_{TC}||\frac{C_{PZ}}{{k_{T}}^{2}}\right)\right]}}\\ & =\frac{f_{R}}{\sqrt{\left(1-k_{CL}\right)+\frac{k_{CL}C_{PZ}}{L_{TC}{k_{T}}^{2}+C_{PZ}}}}\equiv f_{R}k_{F}, \end{array}$$

with a quality factor, $Q_{R}’$, of

(12) $$Q_{R}’=\frac{1}{R_{R}’}\sqrt{\frac{L_{C}’}{C_{L0}’+(C_{LC}’⊕C_{PZ})}}=R_{T}\sqrt{\frac{C_{T}}{L_{T0}+\left(L_{TC}||\frac{C_{PZ}}{{k_{T}}^{2}}\right)}}.$$

In this paper, “⊕” indicates the electrical series combination of capacitors (which are mathematically parallel). 

**Figure 6:**
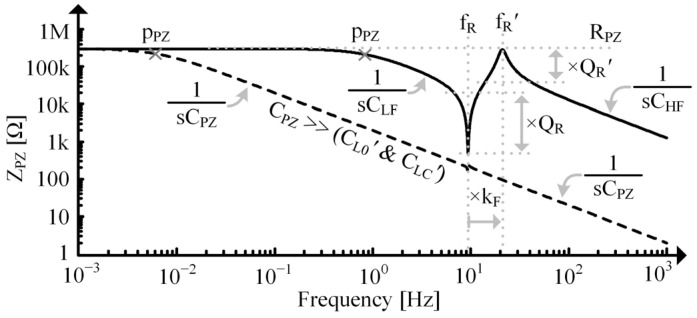
Simulated impedance vs. frequency for a single tone PT on a logarithmic scale.

Below $f_{R}$, $L_{C}’$ shorts, so $C_{PZ}$ and $C_{LC}’$ dominate:

(13) $${Z_{PZ}|}_{P_{PZ}<f_{0}<f_{R}}\approx \frac{1}{sC_{LF}}=\frac{1}{s(C_{LC}’+C_{PZ})},$$

until frequency drops low enough for them to be open relative to $R_{PZ}$, which occurs at $p_{PZ}$, shown in Figure 6. Past $f_{R}’$, capacitors are shorted and $L_{C}’$ is open, so $Z_{PZ}$ is dominated by the series combination of $C_{LC}’$ and $C_{L0}’$ in parallel with $C_{PZ}$:

(14) $${Z_{PZ}|}_{f_{0}>f_{R}’}\approx \frac{1}{sC_{HF}}=\frac{1}{s\left[\left(C_{LC}’⊕C_{L0}’\right)+C_{PZ}\right]}.$$

Note that $f_{R}’$ and $Q_{R}’$ depend on $C_{PZ}$. Specifically, $C_{PZ}$ affects $C_{LC}’$ (or $L_{TC}$), indicating that it directly alters the spring’s mechanical stiffness [9]. $f_{R}$ and $Q_{R}$, however, are independent of the ED. 

$Z_{PZ}$’s response across frequency is shown in Figure 6, but note that each attribute of the response is variable. $f_{R}$ and $f_{R}’$ will be more separated at large $k_{CL}$s and come closer together as $k_{CL}$ reduces. As $C_{PZ}$ gets larger relative to $C_{L0}’$ and $C_{LC}’$, it will dominate the response, and the prominence (i.e., quality factors) of and spacing (i.e., $k_{F}$, shown in (11)) between the $f_{R}$ and $f_{R}’$ peaks will reduce. When $f_{R}$ and $f_{R}’$ get too close together, $Z_{PZ}$’s peaks may no longer correspond to $f_{R}$ and $f_{R}’$.

If $C_{PZ}$ is very small relative to $C_{L0}’$ and $C_{LC}’$, Q and $k_{F}$ will increase until limited by other factors such as mechanical impedances, $k_{T}$, and $k_{CL}$, and $f_{R}’$’s expression reduces to:

(15) $$f_{R}’{|}_{C_{PZ}\ll \left({C_{LC}}^{’}\&{C_{L0}}^{’}\right)}\approx \frac{1}{2\pi \sqrt{L_{C}’C_{L0}’}}=\frac{1}{2\pi \sqrt{C_{T}L_{T}(1-k_{CL})}}.$$

Using (15), $k_{CL}$ can be approximated as:

(16) $${\left.k_{CL}\right|}_{C_{PZ}\ll \left({C_{LC}}^{’}\&{C_{L0}}^{’}\right)}\approx 1-{\left(\frac{f_{R}}{f_{R}’}\right)}^{2}=1-{\left(\sqrt{1-k_{CL}}\right)}^{2},$$

which is sometimes used for PZ resonators [25,29]. This is useful but may not be applicable in many other contexts as the condition may not be satisfied.

The Norton and Thevenin impedances of Figure 3 and Figure 4 are equal to $Z_{PZ}$. The Norton equivalent current source, $i_{NO}$, is equal to the current through a short circuit (SC) across the output, $v_{PZ}$, which in this case, is equal to the current through $C_{LC}’$ [28]. With the output shorted, $L_{C}’$, $R_{R}’$, and $C_{L}’$ are in series. Using the translations in Section 2.2, the current through that series combination is proportional to $v_{T}$ by $k_{T}$:

(17) $$\begin{array}{cc} i_{NO}=i_{PZ(SC)} & =\left(\frac{v_{S}}{Z_{I\left(SC\right)}}\right)\left(\frac{C_{LC}’}{C_{LC}’+C_{L0}’}\right)=k_{T}v_{T\left(SC\right)}k_{CL}\\ & =\left(\frac{i_{S}}{k_{T}}\right)\left[\frac{1}{R_{R}’+sL_{C}’+\left(\frac{1}{sC_{LC}’}||\frac{1}{sC_{L0}’}\right)}\right]k_{CL}. \end{array}$$

The current through $C_{LC}’$ is a current divided fraction, $k_{CL}$, of $C_{L}’$, as seen in (6). The Thevenin equivalent voltage source is related to $i_{NO}$ and $Z_{PZ}$:

(18) $$v_{TH}=i_{NO}Z_{PZ}.$$

### 2.4. Additional Resonant Tones

A PT can resonate at multiple frequencies depending on its geometry. The shape it deforms into at each resonant frequency is known as a mode of vibration, but in the context of electrical engineering, it adds a tone to the frequency spectrum [30]. 

**Figure 7:**
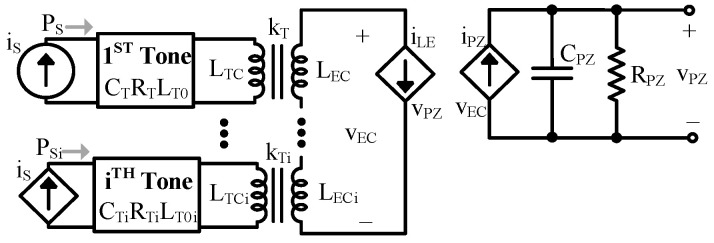
Complete model with additional tones.

Each tone adds resonance in the same way as the fundamental, so adding LCR loops coupled to the ED via transformers accounts for them, as shown in Figure 7. Note that the current/force source in the additional tones is equal to the fundamental’s source, and that the mechanical parameters, $k_{T}$, and $k_{CL}$ are likely different for each tone [10].

**Figure 8:**
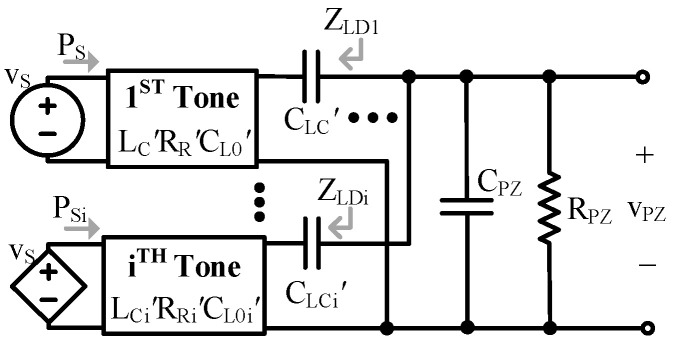
Electrical model with additional tones.

While the $L_{EC}$ components of each tone experience the same current, the different $k_{T}s$ mean different current is induced to each tone. The tones’ unique impedances also mean the ac part of $i_{LE}$ affects each tone’s voltage differently. When translated, each tone adds an LCR branch which couples to the electrical load via $C_{LC}’$, as shown in Figure 8. In this case, each branch has the same voltage, but the voltage coupled to each tone is unique because the $C_{LC}’$s are different. 

**Figure 9:**
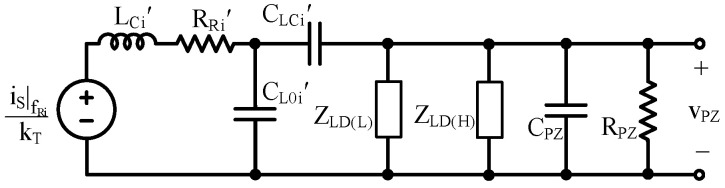
Electrical model of the ith tone with simplified tonal loading.

Interestingly, this means that tones add load capacitance relative to other tones. In other words, if looking at a particular tone, i, any tone greater or smaller than i will add to i’s load capacitance, as shown in Figure 9, (20) and (21). 

For tones lower than i, $L_{Cn}’$ is open circuited (OC), so the impedance of those tones is dominated by the series combination of $C_{LCn}’$ and $C_{L0n}’$ of each tone:

(19) $$Z_{LDi}\approx \frac{1}{s\left(C_{LCi}’⊕C_{L0i}’\right)}.$$

The impedance of each tone appears parallel to each other at the output, so the combined load impedance they contribute is:

(20) $$Z_{LD\left(L\right)}={Z_{LD}|}_{tones<i}=Z_{LDi}||\cdots ||Z_{LD\left(i-1\right)}\approx \frac{1}{s\sum_{n=1}^{i-1}\left(C_{LCn}’⊕C_{L0n}’\right)}.$$

For tones higher than i, $L_{Cn}’$ is a short, so those tones contribute a load impedance, $Z_{LD\left(H\right)}$, dominated by $C_{LCn}’$ of each tone:

(21) $$Z_{LD\left(H\right)}={Z_{LD}|}_{tones>i}\approx \frac{1}{s\sum_{n=i+1}^{\infty }\left(C_{LCn}’\right)}.$$

## 3. Electromechanical Coupling

### 3.1. Coupling Coefficients

The goal of a PT coupling coefficient is to express the fraction of energy that reaches the ED from the MD (or vice versa). The energy that does not reach the ED is not necessarily lost, as it could remain stored in the MD [31]. To determine the fraction of energy in the MD that reaches the ED, two components of cross-domain energy transfer need to be discussed. First is the fraction of mechanical energy that is available to the ED. The second arises from the fact that only a fraction of that available energy can be captured and used by the ED.

The fraction of mechanical energy available to the ED is directly related to $k_{CL}$ since it represents the PT’s inherent ability to convert energy between domains. To capture energy in the ED, though, an electrical load, such as $C_{PZ}$, needs to be present, but this reduces the fraction of energy available to the ED since impedance is added to the coupling network [31]. In other words, $k_{CL}$ is effectively reduced in the presence of an electrical load. In Figure 4, the series combination of $C_{PZ}$ and $C_{LC}’$ forms a current divider with $C_{L0}’$, so only a fraction of the power entering the capacitor network, $P_{LT}$, reaches the EM branch, $P_{EM}$, thus less mechanical energy is available to the ED. This effect is captured by the mechanical coupling coefficient:

(22) $$k_{CM}=\frac{i_{EC}}{i_{VS}}=\frac{\frac{1}{sC_{L0}’}}{\frac{1}{sC_{L0}’}+Z_{EQ}}=\frac{\frac{1}{sC_{L0}’}}{\frac{1}{sC_{L0}’}+\left[\frac{1}{sC_{LC}’}+\left(\frac{1}{sC_{PZ}}||R_{PZ}\right)\right]}.$$

Mechanically, $C_{PZ}$ effectively increases the spring’s stiffness (i.e., reduces $L_{TC}$), reducing the energy it can store for a given force packet, meaning less is available for coupling [9,31]. $k_{CM}$ is less than or equal to $k_{CL}$. 

When OC, the energy stored in $C_{PZ}$ can be considered as having reached the ED since this is energy that the ED can use. Because $L_{TC}$ is finite, the transformer in Figure 3 requires some energy to transform voltage when loaded. This energy does not reach the ED, but is also not lost, it simply remains in the MD [26]. In Figure 4, $C_{PZ}$ forms a voltage divider with $C_{LC}’$, so only a fraction of $P_{EM}$ reaches $C_{PZ}$. In other words, some energy remains in $C_{LC}’$ and does not reach $C_{PZ}$. This effect is captured with the electrical coupling coefficient:

(23) $$k_{CE}=\frac{v_{PZ}}{v_{LT}}=\frac{Z_{PZ}’}{\frac{1}{sC_{LC}’}+Z_{PZ}’}=\frac{\frac{1}{sC_{PZ}}||R_{PZ}}{\frac{1}{sC_{LC}’}+\left(\frac{1}{sC_{PZ}}||R_{PZ}\right)},$$

which ideally equals 1. Mechanically, this occurs because the coupled portion of the spring’s stiffness is shared between the MD and ED, so an applied force will result in energy that is partially stored in the coupled mechanical spring, $L_{TC}$, and partially in the “electrical spring”, $C_{PZ}$ [9].

$k_{CL}$ represents the *maximum* fraction of mechanical energy available to the ED. $k_{CM}$ pertains to what fraction of that maximum mechanical energy is *actually* available to the ED when a load is present. $k_{CE}$ pertains to what fraction of the available energy is captured by the ED. Note that $k_{CM}$ and $k_{CE}$ are load dependent. The product of these two is the fraction of mechanical energy that is useable/harvestable in the ED and is referred to here as the PT coupling coefficient:

(24) $$k_{C}=\frac{P_{EC}}{P_{LT}}=k_{CM}k_{CE}.$$

Theoretically, $k_{C}$ cannot be larger than $k_{CL}$, regardless of loading conditions, as $k_{CL}$ is a material limit. 

### 3.2. Coupling Extremes

As $k_{CL}$ decreases, it is harder for actions in the ED to affect motion (i.e., it takes a higher $v_{PZ}$ to affect $v_{LT}$). From this perspective, a small $k_{CL}$ implies a “weakly coupled” PT. This is not to say, though, that the ED cannot have significant effects on motion when $k_{CL}$ is low. If $C_{PZ}$ is small enough, even a small $i_{EC}$ can produce a large $v_{PZ}$. It is important to note that coupling effects are ultimately determined by the combined effects of $L_{T}$, $k_{CL}$, and ${k_{T}}^{2}$ relative to the electrical load. Therefore, a small $k_{C}$, which accounts for all those parameters, always means the PT is weakly coupled.

If $C_{LC}’$ is very small, it becomes more difficult for energy to reach the ED because $C_{PZ}$ would need to be significantly smaller for $k_{CE}$ to approach 1 (assuming $C_{PZ}$ is the only electrical load). When $k_{CE}$ approaches 0, only a small fraction of energy is shared between domains, so the MD and ED are essentially disconnected. A very small $k_{CL}$, though, does not necessarily mean that $C_{LC}’$ is small, as it is also a function of $L_{T}$ and ${k_{T}}^{2}$. $k_{CE}$ approaching 0, however, always means $C_{LC}’$ is much smaller than $C_{PZ}$, so:

(25) $$Z_{PZ}{|}^{k_{CE}\to 0}\to \frac{1}{sC_{PZ}}||R_{PZ}.$$

In other words, $C_{PZ}$ effectively shorts the MD. Since capacitance typically corresponds to large impedances at frequencies of interest for energy harvesting, a Norton equivalent is preferred to model this extreme. This is often performed in the SoA by saying the PT is “weakly coupled” without much further justification, but now (23) and (25) can be used definitively [1,4,7,16,17,18,19,20]. Note that PTs traditionally considered weakly coupled may not actually fulfill this approximation. Note also, that changing the load could change the applicability of the approximation.

If $k_{CL}$ approaches 1, the MD becomes fully connected to the ED, so $Z_{PZ}$ includes $f_{R}$ and $f_{R}’$. $k_{CM}$ approaching 1 means the same thing but also implies $C_{PZ}$ is much bigger than $C_{LC}’$ and $C_{L0}’$. At $f_{R}$, $L_{C}’$ and $C_{LC}’$ effectively short and $C_{L0}’$ opens, so:

(26) $$Z_{PZ}{|}_{f_{R}}^{k_{C}\to 1}\to R_{R}’||R_{PZ}\approx R_{R}’,$$

since $R_{PZ}$ is typically big. $R_{R}’$ is usually small, so for these cases, a Thevenin equivalent would be preferred. At $f_{R}’$, the resonant network exhibits a huge impedance parallel to $R_{PZ}$, so:

(27) $$Z_{PZ}{|}_{f_{R}’}^{k_{C}\to 1}\to R_{PZ}.$$

Since $R_{PZ}$ is normally large, this case is also best modeled with a Norton equivalent. 

### 3.3. Coupling with a Capacitive Load

When a PT is loaded with a capacitor, $C_{LD}$, such that the total output capacitance, $C_{O}$, is:

(28) $$C_{O}=C_{PZ}+C_{LD},$$

and assuming $R_{PZ}$ is very large, an interesting tradeoff exists between $k_{CM}$ and $k_{CE}$. To maximize $k_{CM}$, $C_{O}$ should be much larger than $C_{LC}’$ and $C_{L0}’$, but to maximize $k_{CE}$, $C_{O}$ should be much smaller than $C_{LC}’$ and $C_{L0}’$. So, if $k_{CM}$ is maximized, $k_{CE}$, and therefore $k_{C}$, go to 0, and vice versa. This implies an optimal $C_{O}$ exists to maximize $k_{C}$, $C_{O(MKC)}$.

Using (21)–(24) and approximating $R_{PZ}$ as large, an expression for $k_{C}$ with respect to $C_{O}$ can be made:

(29) $$k_{C}{|}_{f_{R}’}\approx \frac{\left(C_{LC}’⊕C_{O}\right)C_{LC}’}{\left[C_{L0}’+\left(C_{LC}’⊕C_{O}\right)\right]\left(C_{LC}’+C_{O}\right)}.$$

$k_{C}$ is evaluated at $f_{R}’$ as this is the frequency at which the maximum voltage occurs and the frequency the beam freely vibrates at. To find $C_{O(MKC)}$, the derivative of (29) with respect to $C_{O}$ is equated to 0:

(30) $$\frac{dk_{C}}{dC_{O}}\approx -\left(\frac{{C_{LC}’}^{2}\left(C_{L}’{C_{O}}^{2}-{C_{LC}’}^{2}C_{L0}’\right)}{{\left(C_{O}+C_{LC}’\right)}^{2}{\left(C_{L}’C_{O}+C_{L0}’C_{LC}’\right)}^{2}}\right)\equiv 0.$$

Solving for $C_{O}$ in (30) gives the optimal $C_{O}$ to maximize $k_{C}$ for a particular $k_{CL}$:

(31) $$C_{O(MKC)}\approx C_{LC}’\sqrt{\frac{C_{L0}’}{C_{L}’}}=C_{L}’k_{CL}\sqrt{1-k_{CL}}.$$

Plugging (31) back into (29) gives an expression for the maximum PT coupling coefficient that can be achieved for a PT, $k_{C(MAX)}$, with a particular $k_{CL}$ and capacitive load:

(32) $$k_{C\left(MAX\right)}=k_{C}{|}_{C_{O\left(MKC\right)}}\approx {\left(\frac{\sqrt{k_{CL}}}{1+\sqrt{1-k_{CL}}}\right)}^{2}.$$

**Figure 10:**
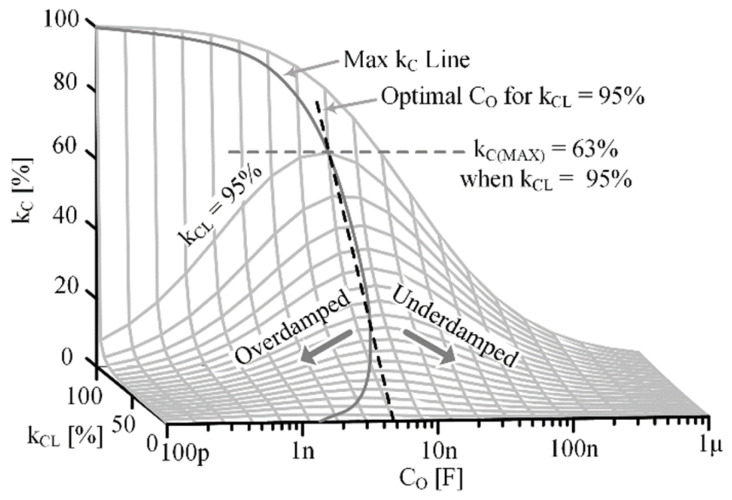
Simulated $k_{C}$ at various $k_{CL}$ and $C_{O}$ combinations.

This highlights the difference between a PT’s intrinsic ability to couple between domains, $k_{CL}$, and the fraction of energy that can be practically captured in the ED, $k_{C}$. It also highlights the effect electrical loading can have on available electrical energy, since in this case, $k_{C(MAX)}$ is always less than $k_{CL}$, unless $k_{CL}$ equals 1. The $k_{C(MAX)}$ line for a given $k_{CL}$ can be seen in Figure 10. If $C_{O}$ is larger than $C_{O(MKC)}$, then the PT’s motion is increased, so more energy is available, but the ability to capture that energy in the ED is decreased, underdamping the system. If $C_{O}$ is smaller than $C_{O(MKC)}$, the ability to capture energy in the ED is increased, but mechanical motion is decreased, overdamping the system. Optimizing $k_{C}$ will always improve electrical energy capture, but as $k_{CL}$ becomes very small (much less than 1% for example), the conditions required to achieve optimization could become impractical.

## 4. Parameter Extraction

The model and analysis presented in the previous sections are applicable to many PT types and dimensions, as discussed in Section 2.1. This section describes a method for extracting each parameter of the model specifically from a real cantilever-style PT with a tip mass to illustrate a way to use the model practically. The methods can be expanded upon in future work to develop extraction methods for other types of PTs and contexts. The method begins by assuming a single-tone PT and then provides techniques for accounting for higher tones.

### 4.1. Mechanical Parameters

The mechanical parameters are arguably the simplest to extract, so they are determined first. If the PT’s tip mass, $M_{T}$, is much larger than the PT’s mass, then [22,23]:

(33) $$C_{T}=M_{T}.$$

**Figure 11:**
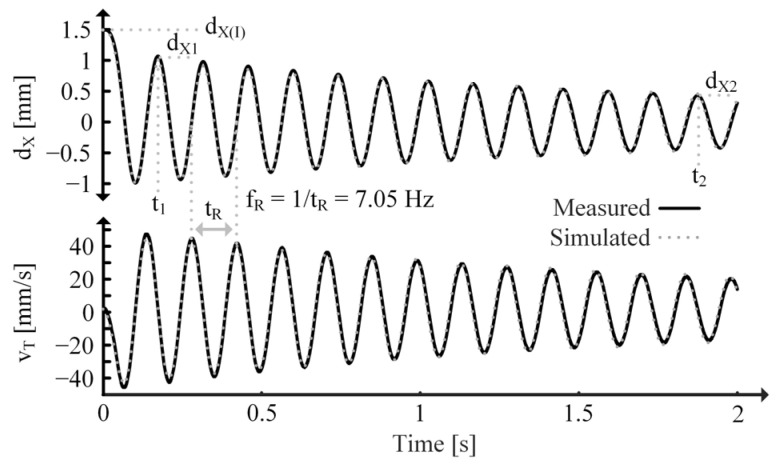
Measured/simulated $d_{X}$ and $v_{T}$ for initially displaced SC PT.

Therefore, $C_{T}$ can be determined by weighing the tip mass. Since the PT and mass form a spring-mass resonator, $L_{T}$ is related to $C_{T}$ by $f_{R}$. To measure $f_{R}$, SC the PT’s output so the ED is not loading the MD and measure the period of $d_{X}$ and/or $v_{T}$ of an initially displaced beam using a displacement sensor, as shown in Figure 11. Measuring the response of an initially displaced beam allows the PT to resonate freely at its $f_{R}$. 

$C_{T}$ and $f_{R}$ can then be used to determine $L_{T}$:

(34) $$L_{T}={\left(\frac{1}{2\pi f_{R}}\right)}^{2}\left(\frac{1}{C_{T}}\right)={\left(\frac{t_{R}}{2\pi }\right)}^{2}\left(\frac{1}{C_{T}}\right).$$

The PT-mass system exhibits damped resonance, so its behavior is of the form:

(35) $$i_{LT}=\frac{d_{X}}{L_{T}}=i_{LT\left(I\right)}e^{-\left(\frac{t}{2R_{T}C_{T}}\right)},$$

where $i_{LT(I)}$ represents the initial force in the spring resulting from the initial displacement, $d_{X(I)}$. By choosing two different points along the $d_{X}$ curve, such as ($d_{X1}$, $t_{1}$) and ($d_{X2}$, $t_{2}$) as shown in Figure 11, two different instances of (35) are determined. Taking the ratio of these instances and rearranging the expression to isolate $R_{T}$ results in:

(36) $$R_{T}=\frac{t_{2}-t_{1}}{2C_{T}ln\left(\frac{d_{X1}}{d_{X2}}\right)}.$$

### 4.2. Electrical Parameters

Extracting the electrical parameters is a little more involved. Recall from Section 2.3 that past $f_{R}’$, $L_{C}’$ is open, so the measured impedance, $Z_{O}$, at these frequencies would be:

(37) $$\begin{array}{cc} Z_{O}{|}_{f_{0}\gg f_{R}’} & \approx \frac{1}{sC_{HF}}=\frac{1}{sC_{PZ}}||\frac{1}{s\left(C_{LC}’⊕C_{L0}’\right)}\\ & =\frac{1}{sC_{PZ}}||\frac{1}{s\left(L_{T}k_{CL}\left(1-k_{CL}\right){k_{T}}^{2}\right)}. \end{array}$$

Note that $C_{PZ}$ cannot be measured directly, but $C_{HF}$ can be.

**Figure 12:**
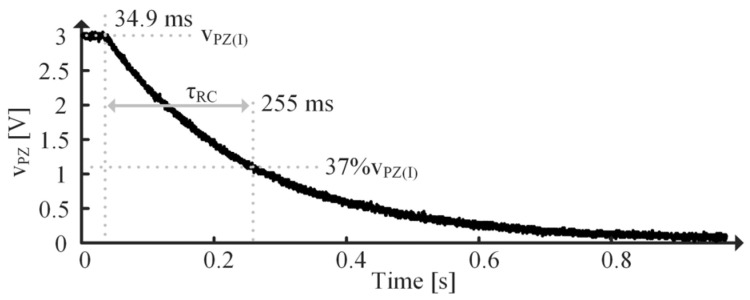
Measured discharge of immobilized PT.

There are two main challenges with using an impedance analyzer (IA) to determine $R_{PZ}:$ output impedance and minimum frequency. $R_{PZ}$ is typically very large (on the order of MΩ), so the IA’s output impedance needs to be larger than that to give an accurate measure of $R_{PZ}$. Plus, since $R_{PZ}$ is so large, $p_{PZ}$ typically occurs around or below 1 Hz, so the IA needs to operate at those frequencies to provide a measurement of $R_{PZ}$. If an IA is on hand which satisfies these requirements, that is the best method for determining $R_{PZ}$. If such an IA is not accessible, the PT’s discharge rate, shown in Figure 12, can be used since a PT’s output is largely capacitive.

Begin by immobilizing the beam—this ensures $Z_{O}$ is dominated by $C_{HF}$ across all frequencies greater than $p_{PZ}$ by forcing $v_{T}$ to 0. Then connect the PT to a power supply set to a particular voltage and allow it to charge. Disconnect the PT from the supply and measure the voltage across the PT’s terminals, $v_{PZ}$, with an oscilloscope. The time it takes for the voltage to drop from the initial voltage, $v_{PZ(I)}$, to 37% of $v_{PZ(I)}$ is one time constant, $\tau_{RC}$. $R_{PZ}$ can be determined using:

(38) $$R_{PZ}=\frac{\tau_{RC}}{C_{HF}}.$$

**Figure 13:**
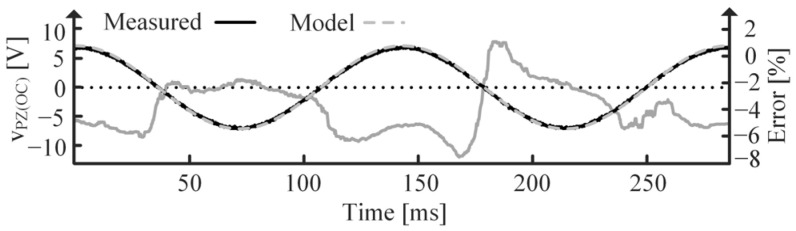
Measured $v_{PZ(OC)}$ and modeled $v_{PZ(OC)}$ after curve fitting with resulting percent error.

Rearranging (37) and substituting in (17) for $k_{T}$ gives an expression for $C_{PZ}$:

(39) $$C_{PZ}=C_{HF}-L_{T}\left(\frac{1-k_{CL}}{k_{CL}}\right){\left(\frac{i_{PZ,PK\left(SC\right)}}{v_{T,PK\left(SC\right)}}\right)}^{2},$$

where $i_{PZ,PK(SC)}$ is the measured, peak, SC current corresponding to a particular measured, peak, SC velocity, $v_{T,PK(SC)}.\;v_{T,PK(SC)}$ can be measured using a $d_{X}$ sensor. There are two unknowns in (39): $C_{PZ}$ and $k_{CL}$, but it is known that $k_{CL}$ is between 0 and 1. To determine $C_{PZ}$ and $k_{CL}$, software aided curve fitting is performed on the OC $v_{PZ}$, shown in Figure 13. Curve fitting computes a selection of variables to yield the least error within given boundary conditions [32,33]. (39) is used as a boundary condition to ensure the results apply to the PT. (39) and $v_{PZ(OC)}$ curve fitting essentially act as two equations to solve for the two unknowns: $C_{PZ}$ and $k_{CL}$. Only $k_{CL}$ is adjusted during fitting since all other parameters have been determined, and because the bounds are well defined, there should only be one unique solution. After $k_{CL}$ is determined from the fit, it is plugged into (39) to find $C_{PZ}$. $k_{T}$ can then be found with (17) using $k_{CL}$, $i_{PZ,PK(SC)}$ and $v_{T,PK(SC)}$. 

The relationship between $k_{CL}$ and $L_{T}$ is described in Section 2.1. To restate, $k_{CL}$ can be used to determine $L_{TC}$ with:

(40) $$L_{TC}=L_{T}k_{CL},$$

and $L_{T0}$ with:

(41) $$L_{T0}=L_{T}-L_{TC}=L_{T}\left(1-k_{CL}\right).$$

### 4.3. Approximating 2nd Tone

As discussed in Section 2.4, higher tones can affect PT behavior, so it can be important to account for them. Approximating the second tone is discussed here, but the methods could be extended to higher tones. Unfortunately, it is difficult to extract these parameters since $C_{T2}$ is not equal to $M_{T}$. 

**Figure 14:**
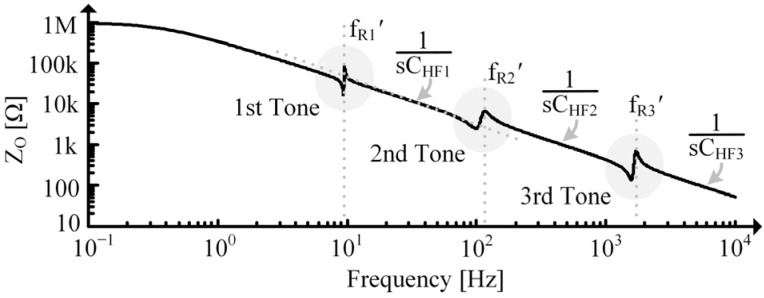
Simulated $Z_{O}$ for multi-tone PT to show $C_{HF}$ measurements.

If operating the PT with a constant applied force of frequency $f_{R}’$, no energy is supplied by the higher tones, so they simply act as capacitive loads. In this case, use $C_{HF1}$, shown in Figure 14, in (39). This way, $C_{PZ}$ will include the capacitive effects contributed by the higher tones, providing the correct loading with respect to the MD but overestimating the ED’s energy storage capacity.

If working with a step function input, the higher tones supply energy to the ED, so it is ideal to account for their full models. While $f_{R2}’$ can be measured, $C_{T2}$ is unknown, so $L_{T2}$ cannot be determined. $C_{HF2}$ and $i_{PZ,PK(SC)}$ can also be measured, but $v_{T,PK(SC)}$ can be difficult to measure since a high-speed, high-sensitivity displacement sensor is required. So, to approximate $R_{T2}$, $C_{T2}$, $L_{T2}$, $k_{T2}$, and $k_{CL2}$, parameter estimation/curve fitting can be used [32,33]. $C_{PZ}$ has a similar limiting condition as before, but this time, the first tone capacitance is accounted for, and more is unknown:

(42) $$C_{PZ}=C_{HF2}-\left(C_{LC1}’⊕C_{L01}’\right)-L_{T2}\left(\frac{1-k_{CL2}}{k_{CL2}}\right){\left(\frac{i_{PZ2,PK\left(SC\right)}}{v_{T2,PK\left(SC\right)}}\right)}^{2}.$$

Note that while curve fitting here should provide a good approximation of the effects of the second tone, since so many variables are being fitted and the limiting conditions are not as rigorous or as well-defined as for the first tone, the resulting second tone parameters may not necessarily correspond to the actual device parameters, and it is possible that there could be more than one unique solution. However, if there is a relationship between $C_{T2}$ and $M_{T}$ and a good $d_{X}$ sensor is on hand, extraction could be performed like what is described in A and B.

## 5. Model Validation

### 5.1. Test Setup

**Figure 15:**
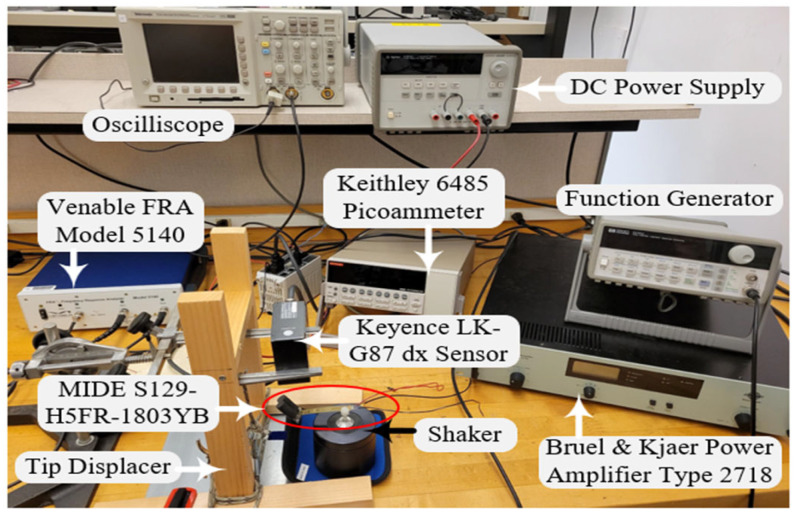
Test setup.

To validate the model described in Section 2, the methods described in Section 4 are performed on the MIDE S129 PT to extract its model parameters using the test setup shown in Figure 15. SPICE simulations are then performed on the model with extracted parameters and the results are compared with measured data. 

The PT is mounted on a Bruel & Kjaer shaker with a 36 g weight attached to its tip. The frequency response analyzer (FRA) is used as the IA. The picoammeter is used to measure $i_{EC}$ and to SC the PT. The oscilloscope measures $v_{PZ}$. The PT is 2.795 × 0.407 × 0.029 in.

### 5.2. Parameter Extraction

The MIDE S129 PT only weighs about 1.4 g, so with a 36 g tip mass, (33) holds true. So, $C_{T}$ is 36 mF (“milli” is used because the SI unit of mass is kg). $d_{X}$ of the PT tip is measured using the Keyence $d_{X}$ sensor shown in Figure 15. While shorted, the PT is bent to an initial displacement, released, and allowed to vibrate freely. Figure 11 shows the measured $d_{X}$ and $v_{T}$, for the PT which is used to extract $L_{T}$ and $R_{T}$. 

**Figure 16:**
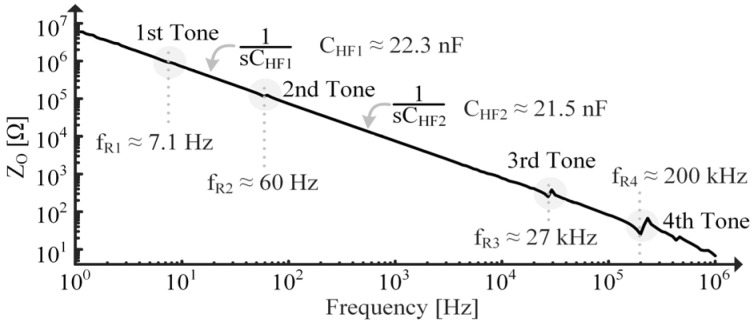
Measured $Z_{O}$ vs. frequency of MIDE S129 with 36 g tip mass using FRA.

$R_{PZ}$ is determined using $\tau_{PZ}$ measured in Figure 12 and the immobilized capacitance of the PT. The PT is immobilized by clamping it to a table and is charged using the power supply shown in Figure 15. The discharge is measured using the oscilloscope in Figure 15. Since the MIDE S129 with a 36 g tip mass has multiple tones, as seen in Figure 16, $C_{HF1}$ is used in (39) and $C_{HF2}$ is used when approximating the second tone parameters. Recall from Section 4.3, though, that using $C_{HF1}$ in (39) means the extracted $C_{PZ}$ includes capacitive loading effects from the higher tones in Figure 16.

**Figure 17:**
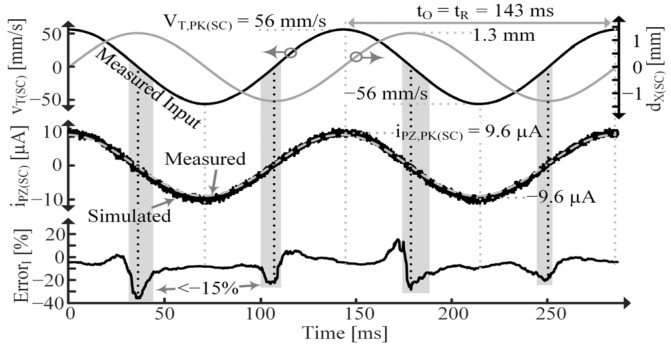
Measured $v_{T(SC)}$, $d_{X(SC)}$, and $i_{PZ(SC)}$ and simulated $i_{PZ(SC)}$ for constantly vibrating PT and average percent error.

The measured $i_{PZ,PK(SC)}$ and $v_{T,PK(SC)}$ are shown in Figure 17. All variables required for (39) have now been measured and/or determined, so curve fitting can commence. The results of the curve fit are shown in Figure 13. All extracted model parameters are shown in Table 1. Plugging these into (23), $k_{CE}$ for this PT loaded by its $C_{PZ}$ is 10%, which is not small enough to assume it is weakly coupled, as (25) shows ($k_{CE}$ would need to be much less than 1%), contrary to what would be performed in the SoA.

### 5.3. Resonant Vibrations

In this section, the PT’s response to a constant applied vibration at $f_{R}’$ is considered and compared to simulated model results. In this case, the force source, $i_{S}$, is not known, but $v_{T(SC)}$ is, so the Norton model shown in Figure 5 is used for simulations.

Consider first the SC case, shown in Figure 17, where measured and simulated $i_{PZ}$ are compared. The error between simulated and measured data is found with:

(43) $$Error=\frac{Simulated-Measured}{Measured},$$

and its average is plotted at the bottom of Figure 17. The areas highlighted in gray indicate where percent error exceeds ±15%. Note that error spikes every time $i_{PZ}$ crosses 0 because if measured data equals 0 and simulated data do not (which happens with the smallest difference in period), error spikes to infinity. 

**Figure 18:**
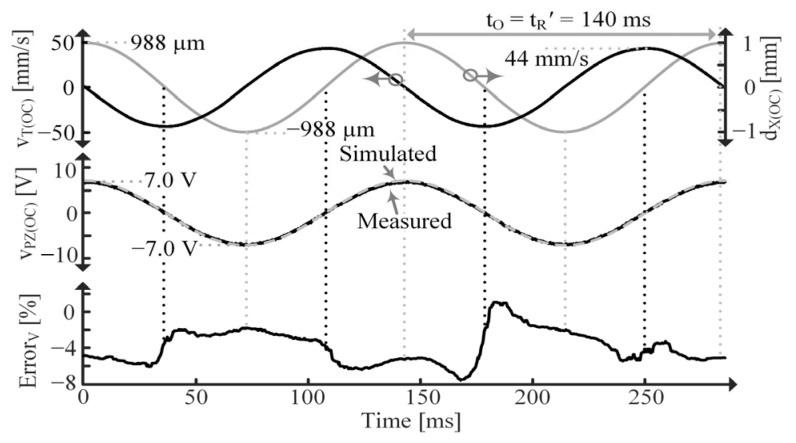
Measured $v_{T(OC)}$, $d_{X(OC)}$ and $v_{PZ(OC)}$ and simulated $v_{PZ(OC)}$ for constantly vibrating PT and average percent error.

Figure 18 shows simulated and measured data for the OC PT. Note the difference in period, $t_{R}$ and $t_{R}’$, between the SC and OC cases, respectively, which results from $C_{PZ}$’s inclusion when OC, as discussed in Section 2.3 [9]. $t_{R}’$ is the period of an $f_{R}’$ vibration. Also note that $i_{PZ}$ is in phase with $v_{T}$ while $v_{PZ}$ is in phase with $d_{X}$ [1,4,7,9,16,17,18,19,20]. ${Error}_{V}$ is smaller than ${Error}_{I}$ because $v_{PZ(OC)}$ is used for curve fitting, so any parameter errors will be more apparent in the $i_{PZ(SC)}$ plots.

**Figure 19:**
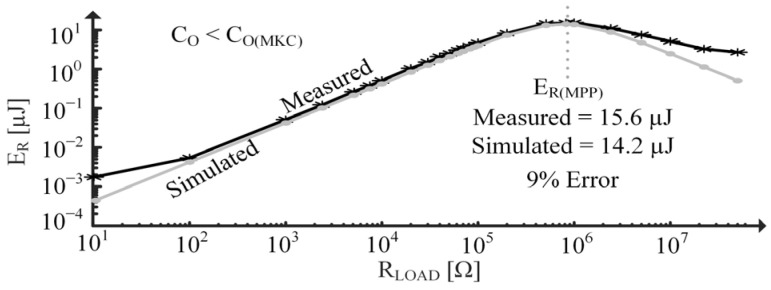
Measured/simulated $E_{R}$ vs. load for constantly vibrating PT.

In Figure 19, resistors are applied across the PT to test the model’s ability to predict accurate responses under various loading conditions. Energy burned by a load resistor, $E_{R}$, is measured across a one-second time interval when the PT experiences the same $v_{T(SC)}$ as in Figure 17. Figure 19 estimates the maximum power point (MPP) resistance to be about the same as what was measured and shows approximately a 9% error between the MPP energies, $E_{R(MPP)}$. Deviations between simulated and measured data at very low $R_{LOAD}$ are attributed to the noise floor limits of the oscilloscope. Similarly, deviation at high $R_{LOAD}$ is most likely due to the limitation of the ammeter.

Error between simulated and measured response likely comes largely from errors in measurements used for parameter extraction. Measuring $C_{HF1}$, for example, can be difficult since it is flanked by resonances, so the impedance line is slightly variable. Small rounding differences when measuring $f_{R}$ can also lead to large error differences since it is squared when calculating $L_{T}$. Certain assumptions/approximations made during parameter extraction, such as neglecting the PT’s mass and approximating impedance as purely capacitive at $C_{HF1}$ can also contribute error. Errors introduced by measurement equipment, such as quantization noise and electronic noise, and the test setup, such as parasitics, can also add to total error.

### 5.4. Step Response

This section discusses the PT’s response to a step input. The PT is bent and clamped to have an initial displacement, then released to resonate freely. This can be modeled in Figure 3 as $i_{S}$ having a constant, non-zero value long enough for the system to reach steady state. At steady state, all the current (force) goes through $L_{T}$ (the spring), which models the PT’s spring storing energy while displaced. To model releasing the PT, drop $i_{S}$ to 0 very quickly. Note this is not a perfect step response as that would require an instantaneous beam release. Instead, there is some “ramp” as $i_{S}$ drops to 0 in a finite amount of time. 

Since the input is approximately a step function, it is ideal to model higher tones. Table 2 shows the approximated 2nd tone parameters found using methods described in Section 4.3. Remember from Section 4.3 that these 2nd tone parameters may not correspond to actual device parameters but provide good approximations for device behavior and are included here to demonstrate the model’s ability to account for higher tones. $C_{PZ}$ also reduces to 19.6 nF since it no longer includes the second tone’s load, per (42). A perfect step function contains many frequencies, but as the step becomes less ideal (i.e., it takes more time to drop to 0), power in the higher frequencies reduces [34]. In this experiment, an ideal beam release was not achieved, so tones higher than the second do not receive enough energy to have significant effects and are thus neglected.

**Figure 20:**
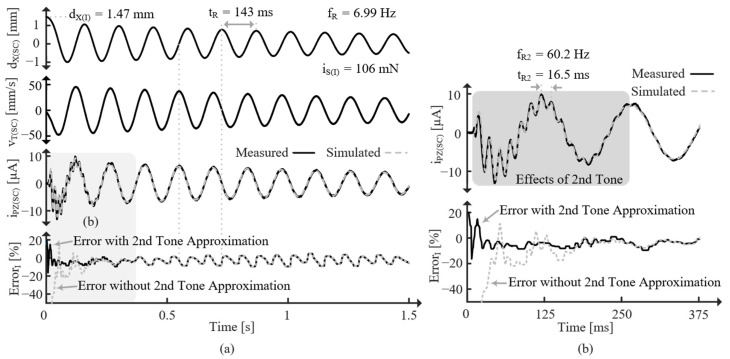
(**a**) Measured $d_{X}$, $v_{T}$, and $i_{PZ}$ for initially displaced SC PT and average percent $i_{PZ}$ error compared with simulations. (**b**) Zoomed in on the gray region of (**a**).

Figure 20 shows the response for the SC PT released from an initial displacement, $d_{X(I)}$, which corresponds to an initial force, $i_{S(I)}$. Note that $i_{S(I)}$ can be determined with (35) because all the current (force) goes through $L_{T}$. Since the PT is SC, it oscillates at $f_{R}$. The effects of the second tone can also be seen, especially in Figure 20b, and its frequency, $f_{R2}$, can be measured. The errors with and without the second tone approximation are shown. After the second tone’s effects subside, the errors are the same and always less than ±10%. However, including the second tone greatly reduces error while its effect is present, indicating the model’s ability to accurately account for higher tones. 

**Figure 21:**
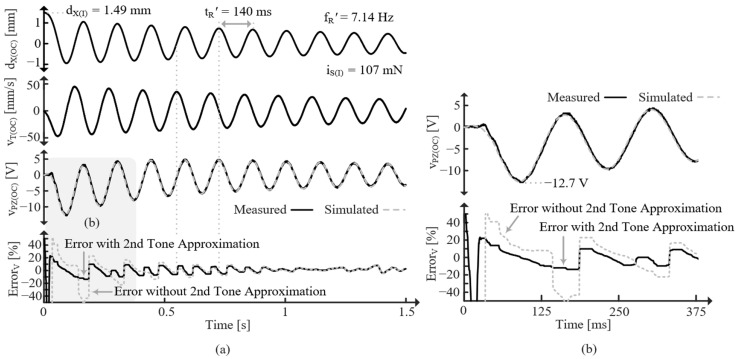
(**a**) Measured $d_{X}$, $v_{T}$, and $v_{PZ}$ for initially displaced OC PT and average percent $v_{PZ}$ error compared with simulations. (**b**) Zoomed in on the gray region of (**a**).

Figure 21 shows the response for the OC PT released from approximately the same $d_{X(I)}$, but this time the PT oscillates at $f_{R}’$ since it is OC. Again, the difference in error with and without the second-order approximation is only present for the first few cycles, but it is significant during those cycles. The reason the error seems to decrease with time is most likely a result of slight frequency error. Interestingly, the simulated voltage is higher (more negative) if the second tone is not accounted for (as is corroborated with the ${Error}_{V}$ plot). This implies that the second tone drains $C_{PZ}$, effectively opposing the first tone [9]. 

**Figure 22:**
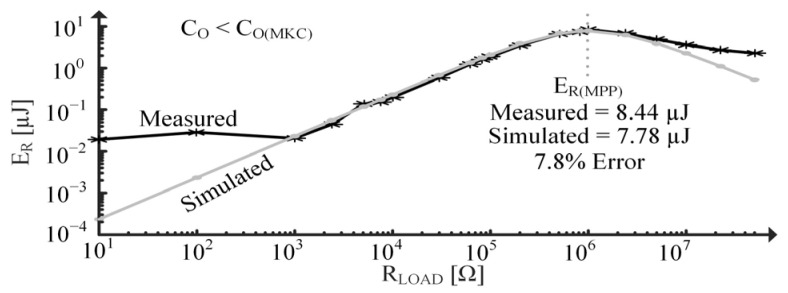
Measured/simulated $E_{OUT}$ vs. load for initially displaced PT.

Figure 22 tests the two-tone model’s ability to predict accurate responses under various loading conditions when the PT is initially displaced. Energy burned by the resistor is measured after the beam has resonated for 9 s with a $d_{X(I)}$ of about 1 mm. The measured and simulated data show good agreement with only about an 8% error and the same MPP $R_{LOAD}$. Excluding the second tone’s effects yields about a 9% error. This difference is likely small because the 9-s window is much larger than the fraction of a second that the second tone is present. If the input were closer to an ideal step function, though, the difference is expected to be larger.

## 6. Conclusions

The complete EM model presented here provides insight into PT energy transfers which was not previously possible by explicitly defining PT parameters not previously represented individually nor completely. The model is generally applicable to many PT types and dimensions and does not rely on coupling assumptions, removing guesswork from designing PT interface circuits. $L_{T}$ is split into its coupled and uncoupled parts, enabling explicit and accurate definition, visualization, and grasp of $k_{CL}$ and $k_{T}$. Incorporating $L_{T}$ into the transformer illustrates its role and related losses in EM energy transfer. Total coupling between domains, limitations to the amount of energy harvestable, and loading effects are shown to be dependent on $k_{CL}$, ${k_{T}}^{2}$, the load, and the PT’s stiffness. It is shown that when $k_{CE}$ approaches 0, the model can be reduced to a current source parallel to $C_{PZ}$ (typical for weakly coupled PTs). When $k_{C}$ approaches 1, a Thevenin equivalent is preferred. 

When constant sinusoidal vibrations are applied to a PT, it is shown that the other tones simply load the fundamental, whereas when an irregular vibration is applied, the other tones can oppose or support energy generation. This model also adequately captures a PT’s impedance behavior making it useful in many contexts, removing the need to switch to different models for different applications, and, in a sense, unifying concepts of various existing PT models into one complete model.

Parameter extraction methods for cantilever style PTs with a tip mass are presented and performed to illustrate a way to use the model practically. Using the extraction methods, designers can effectively and easily model off-the-shelf PTs, enabling easy integration of PTs into SPICE simulations to assist in harvester or interface circuit development. In most cases, less than 5–10% error is observed between measurements and the model with extracted parameters. The model’s ability to capture responses of PTs used as both resonators and energy sources, which are generally accepted in the SoA, further supports it.

The model described here is generalized, but work can be performed to apply it to specific PTs by deriving expressions for each component in Figure 3 in terms of material, dielectric, and piezoelectric constants. Concepts from this paper and [9] and an understanding of transformer turns ratios and mutual inductance can help accomplish this. The resulting expressions would be material and dimension dependent and would provide more insight into the physical parameters contributing to energy generation and loss.

## Figures and Tables

**Table 1:** Extracted Parameters.

Variable	Value
$C_{T}$	36.0 mF
$L_{T}$	13.9 mH
$R_{T}$	32.3 Ω
$k_{CL}$	16.4%
$k_{T}$	993 µ$\frac{Vs}{m}$
$C_{PZ}$	20.4 nF
$R_{PZ}$	11.3 MΩ
$f_{R2}$	60 Hz

**Table 2:** Approximated 2nd Tone Parameters.

Variable	Value
$C_{T2}$	1.0 mF
$L_{T2}$	6.9 mH
$R_{T2}$	54.5 Ω
$k_{CL2}$	45.0%
$k_{T2}$	$133\;µ\frac{Vs}{m}$

## Data Availability

Data are contained within the article.
